# Unlocking PAH Ionization in Negative-Mode ESI Orbitrap MS Using Tetramethylammonium Hydroxide: A Petroleomic Strategy

**DOI:** 10.1021/jasms.5c00170

**Published:** 2025-09-17

**Authors:** Deborah V. A. de Aguiar, Lidya C. da Silva, Iris M. Júnior, Alexandre de O. Gomes, Boniek Gontijo

**Affiliations:** † Laboratory of Chromatography and Mass Spectrometry, Institute of Chemistry, 67824 Federal University of Goiás, Goiânia, GO 59078-970, Brazil; ‡ CENPES, PETROBRAS, Rio de Janeiro, RJ 21941-915, Brazil

**Keywords:** tetramethylammonium hydroxide, petroleum-derived samples, polycyclic aromatic hydrocarbons, electrospray ionization, Orbitrap MS, petroleomics

## Abstract

The selective ionization of cyclopentadiene-derived polycyclic aromatic hydrocarbons (PAHs) in complex matrices remains a persistent challenge in direct ionization mass spectrometry, particularly under atmospheric pressure ionization (API) conditions. In this study, tetramethylammonium hydroxide (TMAH) was evaluated as a solvent modifier to enhance the ionization efficiency of PAHs in negative-ion mode using high-resolution Orbitrap MS. A systematic assessment with equimolar mixtures of neutral and acidic model compounds was performed to elucidate the fundamental effects of TMAH on ion suppression, deprotonation mechanisms, and gas-phase ion chemistry. The incorporation of TMAH significantly improved the detection of weakly acidic and neutral PAHs by promoting efficient gas-phase deprotonation, thus overcoming conventional ESI limitations. To demonstrate the method’s analytical robustness, TMAH-assisted ESI was subsequently applied to crude oil samples, enabling the detection of hydrocarbon species otherwise inaccessible under standard conditions, with a particular focus on low-alkylated fluorene derivatives. These results establish a simple and effective strategy for expanding the analytical scope of ESI-MS toward nonpolar and weakly acidic hydrocarbons, offering a valuable tool for the advanced molecular characterization of complex organic mixtures.

## Graphical abstract

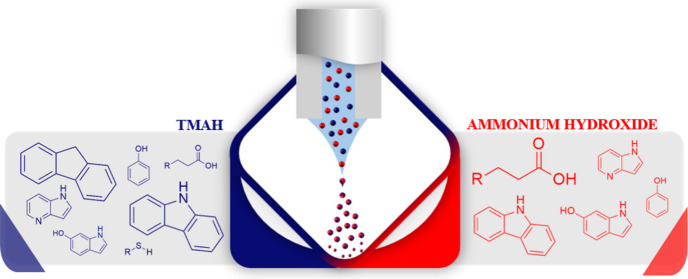

## Introduction

High-resolution mass spectrometry (HRMS) has become a cornerstone technique for the comprehensive molecular characterization of complex mixtures. Its exceptional resolving power and mass accuracy enable the assignment of elemental formulas to tens of thousands of ions in a single analysis.^1–5^ Among available ionization methods, electrospray ionization (ESI) is particularly effective for targeting polar compounds, allowing for directly analyzing heteroatomic species in petroleum-derived samples without requiring prior fractionations.^6–8^

Petroleum-derived samples comprise an extensive array of chemically diverse molecules, predominantly hydrocarbons, accompanied by minor fractions of nitrogen-, oxygen-, and sulfur-containing compounds.^9–11^ Under conventional ESI conditions, the most basic constituents (e.g., pyridinic nitrogen compounds) are typically detected as [M + H]^+^ ions in positive-ion mode, whereas acidic species (e.g., nonbasic nitrogen compounds and carboxylic acids) are observed as [M–H]^−^ ions in negative-ion mode.^12^ However, disparities in the ionization efficiency across various functional groups restrict the detection of several compound classes. As a result, nonpolar species such as hydrocarbons and thiophenic compounds are often underrepresented or undetected.^13^

To overcome these limitations, numerous mass spectrometric strategies have been explored to access compound classes with insufficient proton affinity or acidity for conventional ESI.^14–17^ Among atmospheric pressure ionization techniques, atmospheric pressure photoionization (APPI) and atmospheric pressure chemical ionization (APCI) are currently regarded as the most effective approaches for the characterization of nonpolar compounds in petroleum matrices.^18–23^ Nevertheless, both techniques generate a combination of radical cations and (des)­protonated species, complicating spectral interpretation and hindering confident assignments, particularly in high-complexity samples.^24–28^ Moreover, the elevated operating temperatures associated with these sources may promote dehydrogenation of aromatic compounds, especially in heavier petroleum fractions.^24^

To address the limited ionization of nonpolar compounds by conventional ESI, derivatization strategies have been developed to chemically modify analytes, converting them into more polar species amenable to ionization.^29–34^ For example, Ge et al.^35^ proposed using silver hexafluoroantimonate (AgSbF_6_) as a derivatization reagent for the selective ionization of sulfur-containing compounds in petroleum by ESI-HRMS. In another approach, sulfides were selectively oxidized to sulfoxides using tetrabutylammonium periodate (C_16_H_36_INO_4_), enabling their detection in positive-ion mode via ESI coupled with Fourier transform ion cyclotron resonance mass spectrometry (FT-ICR MS).^36^ Although these methods have proven effective, they are often time-consuming and labor-intensive, prompting the exploration of more practical and efficient alternatives.^13,24,37,38^

In this context, the use of ammonium formiate (HCOONH_4_) has been shown to promote the formation of [M + H]^+^ ions from polycyclic aromatic hydrocarbons (PAHs) in petroleum fractions analyzed by ESI FT-ICR MS.^24^ More recently, tetramethylammonium hydroxide (TMAH, N­(CH_3_)_4_OH), a strong organic base, has attracted attention due to its ability to enhance the ionization of weakly acidic and neutral compounds.^39,40^ TMAH exhibits higher basicity than ammonium hydroxide, allowing the deprotonation of a broader range of compounds with varying p*K*_a_ values. This enhances the ionization of both hydrocarbon and heteroatomic species in negative-ion mode, thereby expanding the analytical window of ESI for complex samples.^13^

The application of TMAH in ESI has demonstrated significant potential for enhancing the ionization efficiency of PAHs, particularly those derived from cyclopentadiene structures. Due to their weak acidity, these compounds exhibit low ionization efficiency under conventional ESI conditions, hindering their detection in complex mixtures. By facilitating gas-phase deprotonation, TMAH significantly improves the formation of [M–H]^−^ ions from these challenging analytes. This strategy enables the direct detection of key PAH species, such as fluorene and its alkylated homologues, without requiring prior chemical derivatization or alternative ionization techniques. Consequently, TMAH-assisted ESI expands the analytical capabilities of mass spectrometry for the molecular characterization of hydrocarbons in complex organic matrices.

This study explores the potential of a TMAH-modified solvent system in ESI to enhance the ionization efficiency of PAHs, with a particular focus on cyclopentadiene-derived structures. Three experimental strategies were employed: (1) evaluating the effect of TMAH on the ionization efficiency of a 50 μmol L^–1^ equimolar model mixture containing 11H-benzo­[*a*]­carbazole (BC), 4H-cyclopenta­[*d,e,f*]­phenanthrene (CyPh), dibenzothiophene (DBT), stearic acid (SA), and benzoic acid (BA); (2) assessing the impact of TMAH on the ionization behavior of chemical constituents in three representative crude oil samples; and (3) demonstrating its capability to detect key hydrocarbon species, such as fluorene and its benzo-derivatives, in complex petroleum matrices. By promoting efficient gas-phase deprotonation, TMAH expands the molecular coverage of ESI-MS, providing a simple and effective strategy for the advanced characterization of hydrocarbons in highly complex organic mixtures.

## Methods and Materials

### Chemicals

The model compounds used in this study included 11H-benzo­[*a*]­carbazole (BC, C_16_H_11_N), 4H-cyclopenta­[*d,e,f]*phenanthrene (CyPh, C_15_H_10_), dibenzothiophene (DBT, C_12_H_8_S), and benzoic acid (BA, C_7_H_6_O_2_), all purchased from Sigma-Aldrich (St. Louis, MO, USA). Stearic acid (SA, C_18_H_36_O_2_) was obtained from Cambridge Isotope Laboratories (Tewksbury, MA, USA). HPLC-grade toluene was sourced from the Tedia Company (Fairfield, OH, USA). HPLC-grade methanol, ammonium hydroxide (NH_4_OH), and tetramethylammonium hydroxide (TMAH, N­(CH_3_)_4_OH) were also purchased from Sigma-Aldrich (St. Louis, MO, USA).

### Sample Preparation

The equimolar mixtures of model compounds were prepared at 50 μmol L^–1^ in a toluene:methanol (1:1, *v/v*) solvent system. Four solutions were prepared: one containing 2% ammonium hydroxide (20 μL NH_4_OH per 1,000 μL methanol) and three others with TMAH at 1%, 2%, and 3% (*v/v*), respectively. These solutions were used to evaluate the effect of the TMAH concentration on the ionization efficiency of the target compounds.

Nine crude oil samples, including five from presalt reservoirs and four from postsalt reservoirs, were provided by the Centre of Research, Development, and Innovation Leopoldo Américo Miguez de Mello (CENPES, Petrobras, Rio de Janeiro, Brazil). Detailed information about these samples is provided in Table S1 (Supporting Information).

In the initial phase, three crude oil samples (**01**, **03**, and **09**) were diluted to a concentration of 1 mg mL^–1^ in toluene. Subsequently, 500 μL of each solution was transferred into 2 mL vials and mixed with 500 μL of methanol containing 2% ammonium hydroxide or TMAH at 1%, 2%, and 3% (*v/v*), resulting in a final concentration of 500 μg mL^–1^ in a toluene:methanol (1:1, *v/v*) solution. For comparative purposes and to evaluate the applicability of TMAH-assisted ESI relative to other atmospheric pressure ionization techniques, the same crude oil samples were also analyzed using APCI and APPI. In these experiments, approximately 1 mg of each sample was dissolved in 1 mL of toluene and subsequently diluted with methanol to obtain final solutions at a concentration of 500 mg L^–1^. Following the evaluation of TMAH’s influence on ionization efficiency, all nine crude oil samples were prepared using a fixed TMAH concentration of 3% *v/v*, following the same protocol.

### Direct Flow Injection High-Resolution MS

Mass spectrometric analyses were performed by direct infusion using a Q-Exactive hybrid quadrupole-Orbitrap mass spectrometer (Thermo Scientific, Bremen, Germany) equipped with a heated electrospray ionization (HESI) source. Samples were introduced via a 500 μL Hamilton syringe by using an integrated syringe pump at a flow rate of 3.0 μL min^–1^. All measurements were conducted in negative-ion mode. The ion source was operated under the following conditions: spray voltage, 3.2 kV; capillary temperature, 275 °C; S-lens RF level, 80; auxiliary gas, 5.0 (arbitrary units); and sheath gas, 2.0 (arbitrary units). The resolving power was set to 140,000 (full width at half-maximum, fwhm, at *m*/*z* 200), which corresponds to approximately 70,000 to 120,000 at *m*/*z* 400, as commonly referenced in petroleum analysis. Full scan mass spectra were acquired over the *m*/*z* range of 100–400 for the equimolar model compound mixture and *m*/*z* 150–1200 for crude oil samples. For comparison purposes only, three representative crude oils (**01**, **03**, and **09**) were also analyzed by APCI and APPI on a 7 T SolariX 2XR FT-ICR MS instrument (Bruker Daltonics, Bremen, Germany) to contrast TMAH-assisted ESI with other API techniques. Samples were infused at 500 μL h^–1^, acquiring 300 scans over *m*/*z* 150–1200. For APCI, a corona current of 2000 nA and a vaporizer temperature of 300 °C were applied. These measurements were performed solely to provide a molecular coverage comparison and to highlight the complementarity of the techniques.

### Mass Calibration and Data Processing

All mass spectra were processed using Composer software (version 1.5.3, Sierra Analytics, CA, USA), which was also employed for the molecular formula assignment of the detected ions. Assigned compounds were categorized based on heteroatom class (type and number of heteroatoms), double bond equivalent (DBE), and degree of alkylation (carbon number). For crude oil samples, molecular formula assignment was performed within the *m*/*z* range of 150–1200, using the following atom constraints: ≤120 carbon atoms, ≤240 hydrogen atoms, ≤2 nitrogen atoms, ≤4 oxygen atoms, and ≤1 sulfur atom. The assignment algorithm followed standard hydrocarbon rules to ensure chemically plausible formulas and employed a mass error tolerance of 1 ppm, applying the walking calibration approach. All assigned molecular formulas were exported to Microsoft Excel and imported into Origin 2018 (OriginLab Corporation, Northampton, MA, USA) for further data analysis and visualization.

## Results and Discussion

### Effect of TMAH Concentration on Model Compounds

Initial experiments were performed to investigate the effect of TMAH concentration on ESI (−) Orbitrap MS analysis of the equimolar model compound mixture. The mass spectra obtained under each condition are shown in Figure 1 (**A**), while the bar plots in panels (**B**) and (**C**) provide a clearer illustration of how each dopant influences the total ion abundance of the model compounds. The addition of ammonium hydroxide as a dopant in the electrospray solvent notably improved the ionization of benzoic acid (BA) and benzo­[*a*]­carbazole (BC), while stearic acid (SA) exhibited a markedly lower abundance. These observations align with previous reports demonstrating that carboxylic acids and nonbasic nitrogen compounds ionize efficiently under basic ESI conditions, which may contribute to the observed ion suppression of other species in the mixture.^11,41^

**Figure 1:**
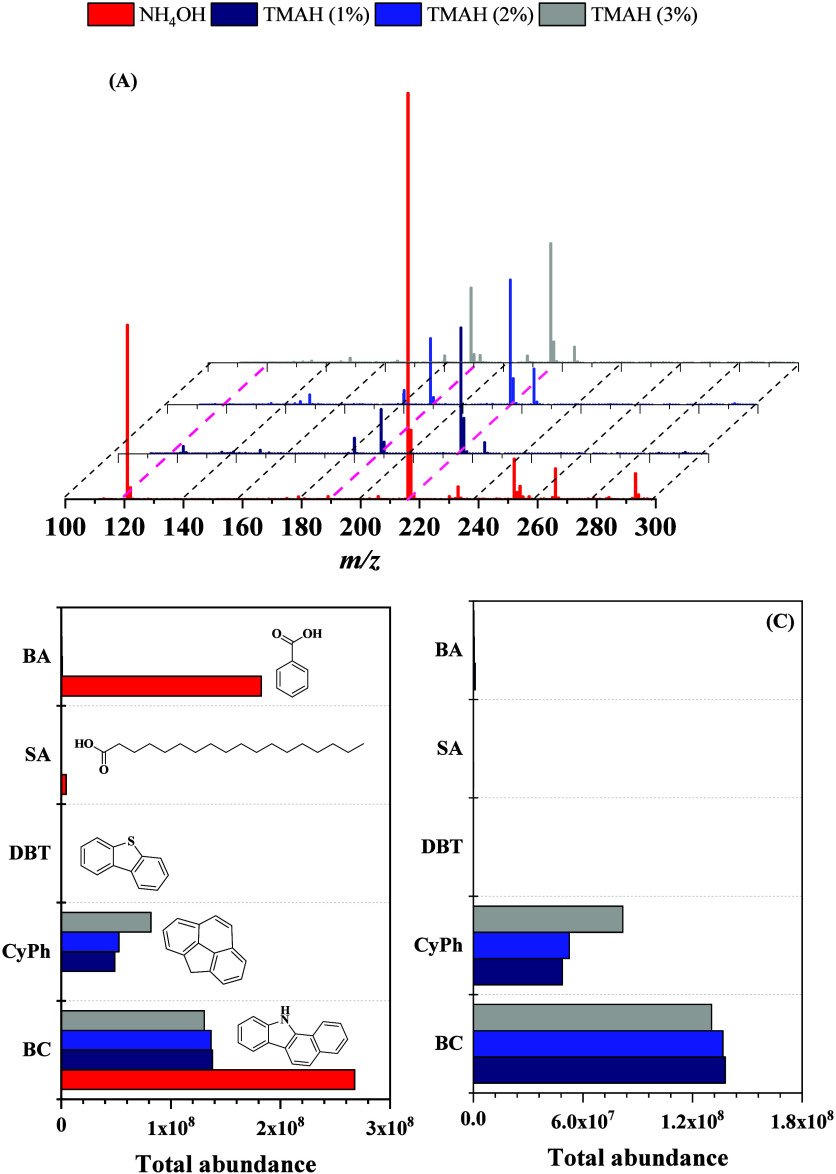
Effect of TMAH concentration on the ionization efficiency of an equimolar model compound mixture analyzed by ESI (−) Orbitrap MS. (**A**) Mass spectra of the equimolar model compound mixture acquired by ESI (−) Orbitrap MS under different dopant conditions: 2% ammonium hydroxide (NH_4_OH, red) and tetramethylammonium hydroxide (TMAH) at 1% (dark blue), 2% (light blue), and 3% (gray). (**B**) Total ion abundance of benzoic acid (BA), stearic acid (SA), dibenzothiophene (DBT), 4H-cyclopenta­[*d,e,f*]­phenanthrene (CyPh), and 11H-benzo­[*a*]­carbazole (BC) in an equimolar mixture under varying ionization conditions: 2% ammonium hydroxide (NH_4_OH) and TMAH at 1%, 2%, and 3% (*v/v*). (**C**) Magnified view of the ion responses under TMAH conditions, highlighting the relative enhancement or suppression of each compound with increasing TMAH concentration.

Ion suppression is commonly attributed to surface charge competition during charged droplet formation, a phenomenon exacerbated when the analyte concentration exceeds the charge capacity of the electrospray droplets.^42,43^ In the ESI process, ionization involves two principal steps: droplet formation and ion desorption into the gas-phase. Analyte ions may be produced through mechanisms such as the ion evaporation model, where ions are expelled from droplets due to surface charge accumulation, or the charged residue model, in which complete solvent evaporation leaves behind the charged analyte.^44,45^ Introducing TMAH as a dopant was essential for mitigating the dominant ionization of strongly acidic compounds, enabling the detection of 4H-cyclopenta­[*d,e,f*]­phenanthrene (CyPh), which was not observed when ammonium hydroxide was used alone. Increasing the TMAH concentration reduced BC signal (factor of 0.94) and a substantial enhancement in CyPh ion abundance (factor of 1.68).

The enhanced ionization of CyPh with TMAH is primarily attributed to its distinct gas-phase properties. Aromatic hydrocarbons such as CyPh, containing cyclopentadienyl moieties, exhibit weak acidity in solution but possess significantly enhanced gas-phase acidity. This occurs because deprotonation at sp^3^ carbon leads to the formation of resonance-stabilized aromatic carbanions, which are efficiently ionized in the presence of TMAH.^13^

In contrast, carboxylic acids such as BA and SA exhibit reduced gas-phase acidity relative to their solution-phase behavior due to the absence of solvent-mediated stabilization of the carboxylate anion (RCOO^–^). This diminished acidity results in a lower ionization efficiency under gas-phase conditions. Additionally, TMAH can interact with carboxylic acids via ion-pair formation, wherein the tetramethylammonium cation (TMA^+^) associates with the carboxylate anion to form species such as [RCOO^–^ + TMA^+^]. Although this interaction stabilizes the anionic form, it may also impede the formation of free carboxylate ions, thereby reducing their effective ionization.^46,47^ Consequently, while TMAH enhances the ionization of compounds like CyPh, it may suppress the ionization of carboxylic acids, emphasizing the critical role of gas-phase ion chemistry and dopant selection in optimizing the electrospray ionization performance.

### Ionization Enhancement in Complex Samples with TMAH

To evaluate the impact of TMAH on the ionization of complex mixtures, three representative crude oil samples (**01**, **03**, and **09**) were analyzed at varying dopant concentrations. Compared with ammonium hydroxide, TMAH substantially increased spectral richness, particularly by expanding the detectable mass range toward lower *m*/*z* values. This enhancement reflects TMAH’s ability to facilitate the ionization of structurally diverse and less acidic compounds that are typically overlooked by conventional ESI conditions.^48–50^

Additional insights into these effects are revealed by the compound class distribution profiles presented in Figure 2. Under ammonium hydroxide conditions, crude oil spectra were dominated by nitrogen- and oxygen-containing species (N and O_2_ classes), with crude oil **09** showing elevated O_2_-class abundance due to its higher total acid number (TAN), as expected for postsalt oils. In contrast, the addition of TMAH shifted the ionization profile by markedly increasing the detection of hydrocarbon-class (HC) compounds, previously suppressed due to their low acidity, while reducing the relative contribution of the O_2_ species. This shift was driven by selective enhancement of weakly acidic hydrocarbons and TMA^+^ interactions with carboxylates, rather than a global suppression effect.^46,47^

**Figure 2:**
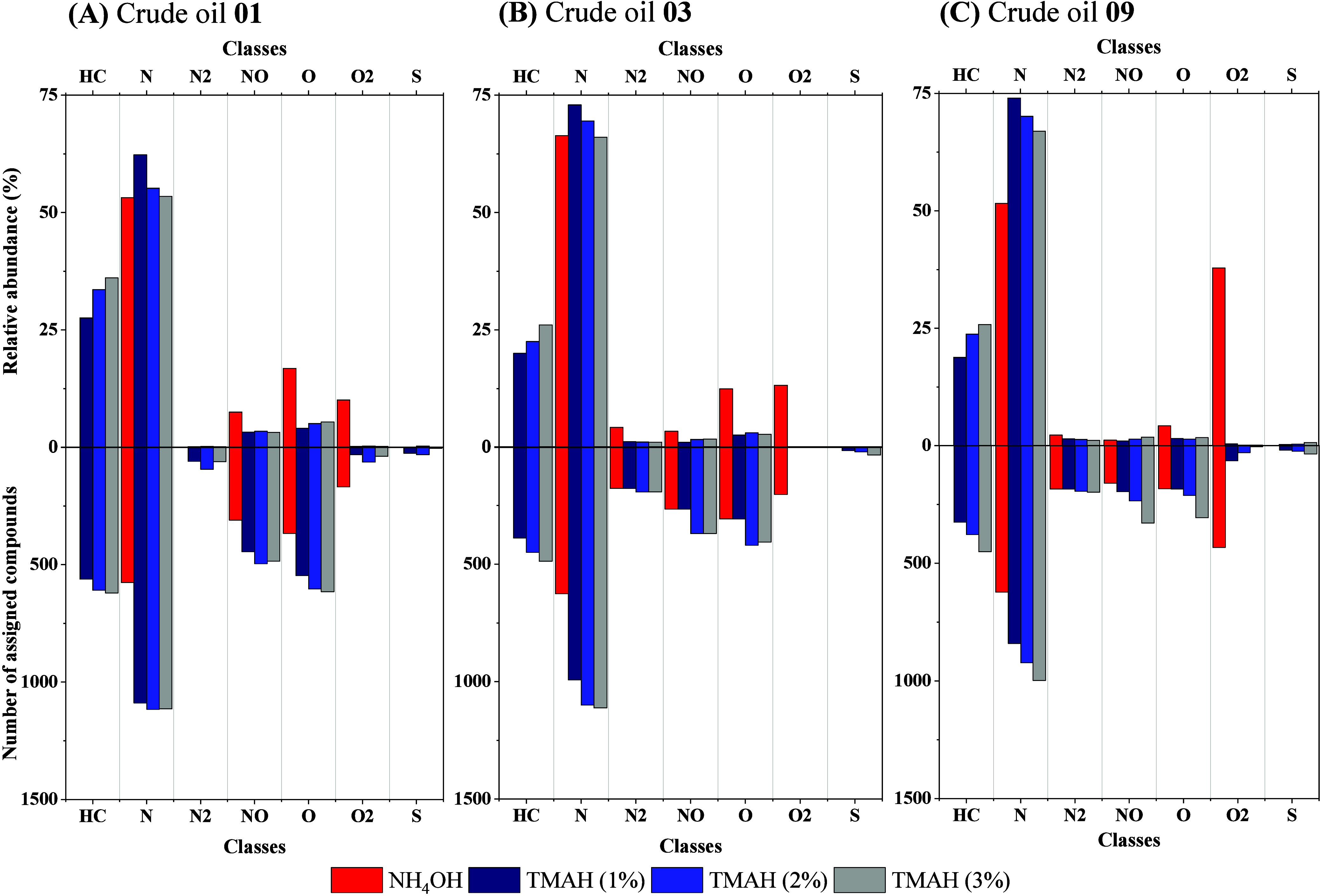
Class distribution of compounds detected in the (**A**) Crude oil **01**, (**B**) crude oil **02**, and (**C**) crude oil **03** analyzed by negative-ion mode ESI Orbitrap MS using 2% ammonium hydroxide and TMAH at 1%, 2%, and 3% (*v/v*).**Top panels:** Relative abundance of compound classes, including hydrocarbons (HC), nitrogen-containing (N, N_2_), oxygen-containing (O, O_2_), nitrogen–oxygen (NO), and sulfur-containing (S) species. **Bottom panels:** Number of assigned molecular formulas per class. Results highlight compositional shifts associated with the TMAH concentration, including enhanced ionization of HC species and suppression of the O_2_-class compounds.

Although hydrocarbons are generally not readily ionized by ESI, the experimental conditions employed in this study enabled their detection as [M–H]^−^ ions. Aromatic hydrocarbons with an sp^3^ carbon, particularly those containing multiple fused rings, were effectively ionized due to the formation of resonance-stabilized aromatic carbanions. This behavior is consistent with prior studies demonstrating that ESI can detect polycyclic aromatic hydrocarbons (PAHs) under specific conditions that favor gas-phase deprotonation.^51,52^

Beyond hydrocarbons, the use of TMAH also reshaped the ionization profiles of moderately acidic compound classes. Species such as N_2_, NO, and O, which are typically abundant under ammonium hydroxide conditions, showed reduced relative abundances with TMAH, even though the total number of assigned molecular formulas increased. This shift reflects a selective enhancement of weakly acidic compounds, broadening the compositional coverage rather than simply redistributing signal intensity. Additionally, sulfur-containing species (S class) became detectable with TMAH, albeit at low relative abundance (<3%), further demonstrating its capacity to reveal low-polarity components within complex mixtures.

To further investigate the compositional nuances enabled by TMAH, Figure S2 provides a detailed visualization of double bond equivalent (DBE) versus carbon number distributions for selected compound classes. The color-mapped plots compare the molecular profiles obtained with 2% ammonium hydroxide and 3% TMAH, revealing distinct differences in the types and structural diversity of the ions detected.

For the N class, TMAH enabled the detection of compounds spanning a wider DBE range (6–25) and carbon numbers (C_18_–C_82_), compared to ammonium hydroxide, which primarily ionized species within DBE 6–21 and C_17_–C_62_. Under ammonium hydroxide conditions, the most intense signals corresponded to carbazole (DBE 9), benzocarbazole (DBE 12), and dibenzocarbazole (DBE 15), moderately acidic nitrogen-containing species.^11^ In contrast, TMAH provided access to a broader molecular window, encompassing these compounds and enabling the ionization of additional nitrogen-containing species across an extended DBE and carbon range. Moreover, TMAH also demonstrated an improved ionization efficiency for other moderately acidic classes, including N_2_, NO, and O.

This broader ionization window is particularly valuable for the characterization of PAHs, as illustrated in Figure 3. Under TMAH-assisted ESI conditions, the detected species are primarily concentrated at DBE values of 9, 12, and 15, which could correspond to fluorene, benzofluorene, and dibenzofluorene derivatives, well-known molecular markers in petroleum geochemistry.^53^ Notably, ESI (−) preferentially accessed compounds within this narrower DBE range, indicative of low-alkylated PAHs with sp^3^-hybridized frameworks. The corresponding carbon number distributions, initiating at C_13_, C_17_, and C_21_ for DBE 9, 12, and 15, respectively, reinforce this interpretation. The sequential addition of three DBE units and four carbon atoms between these groups is consistent with the stepwise fusion of the aromatic ring, supporting the presence of base molecular formulas such as C_13_H_10_, C_17_H_12_, and C_21_H_14_, respectively.

**Figure 3:**
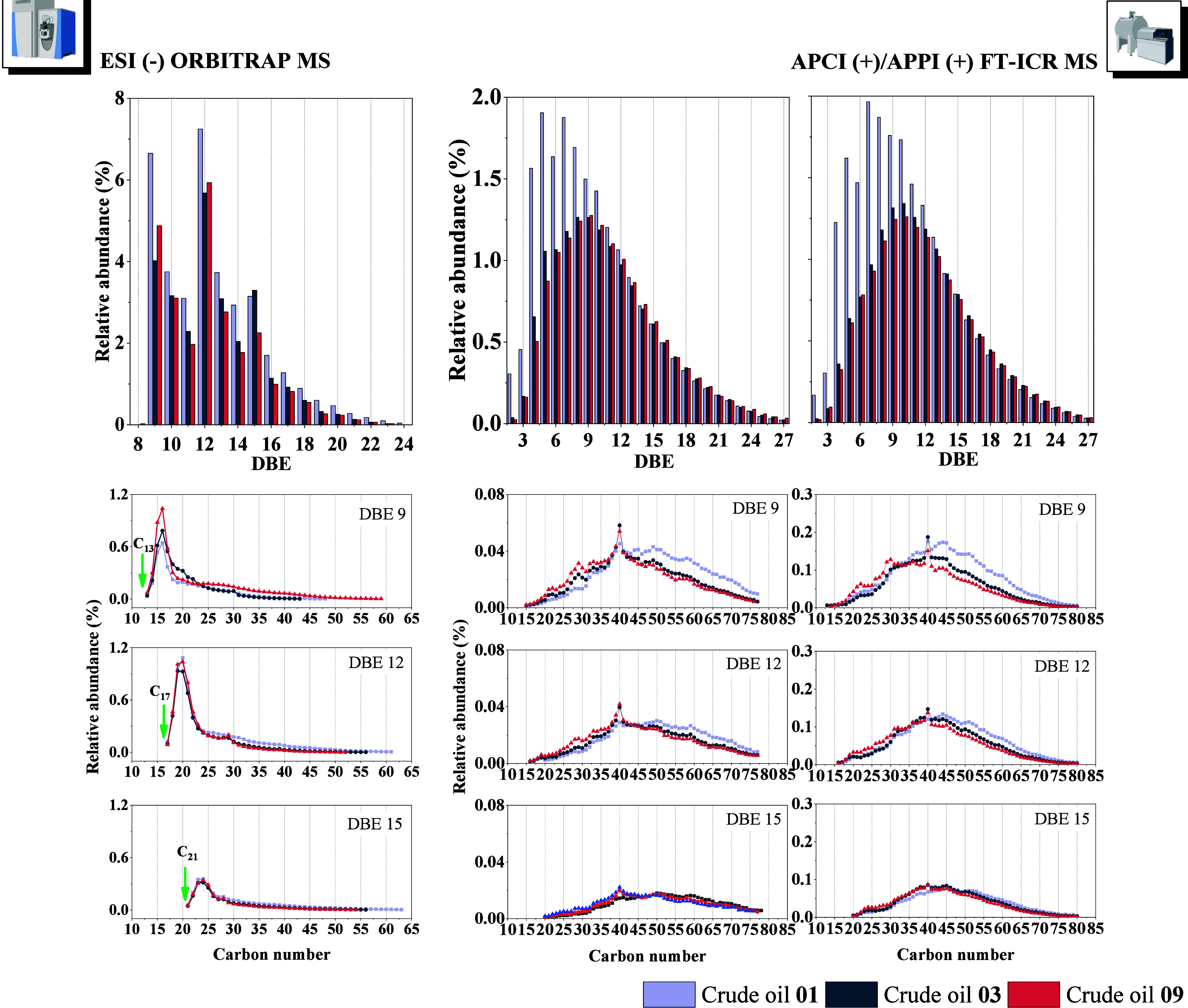
Comparison of DBE and carbon number distributions of hydrocarbon species in three crude oil samples (Crude Oils **01**, **03**, and **09**) analyzed by ESI (−) Orbitrap MS using 3% TMAH (left) and by APCI (+) and APPI (+) FT-ICR MS (middle and right, respectively). The top row shows DBE distribution profiles, while the lower panels present carbon number distributions for the selected DBEs (DBE 9, 12, and 15).

To place these findings in a broader analytical context, we also analyzed the same crude oil samples by APCI (+) and APPI (+) FT-ICR MS. These techniques yielded broader DBE distributions and a notable enrichment of species around C_40_, characteristic of high-mass, highly conjugated PAHs. Ionization in APCI and APPI primarily occurs through radical or charge-exchange pathways, which are particularly effective for condensed aromatic systems, especially in the presence of aromatic solvents such as toluene.^54,55^ Such molecular discrimination underscores the complementarity of TMAH-assisted ESI (−) with traditional atmospheric pressure ionization techniques, offering a targeted and orthogonal strategy for the characterization of PAHs in complex petroleum matrices.

### Analytical Application: Geochemical Insights

To assess the potential of TMAH-assisted ESI for geochemical differentiation, we analyzed nine crude oils, five from presalt and four from postsalt reservoirs, using 3% TMAH as a dopant. Although initial class-level distributions of hydrocarbons (HC), nitrogen- (N), and oxygen-containing (O) compounds (Figure S3) did not yield clear separation between the two groups, the ternary diagram in Figure 4 (**A**) provided a more refined compositional perspective. Presalt oils tended to cluster in regions dominated by hydrocarbon species, while postsalt samples showed increased contributions from N- and O-containing classes, reflecting underlying differences in thermal maturity and organic matter input.

**Figure 4:**
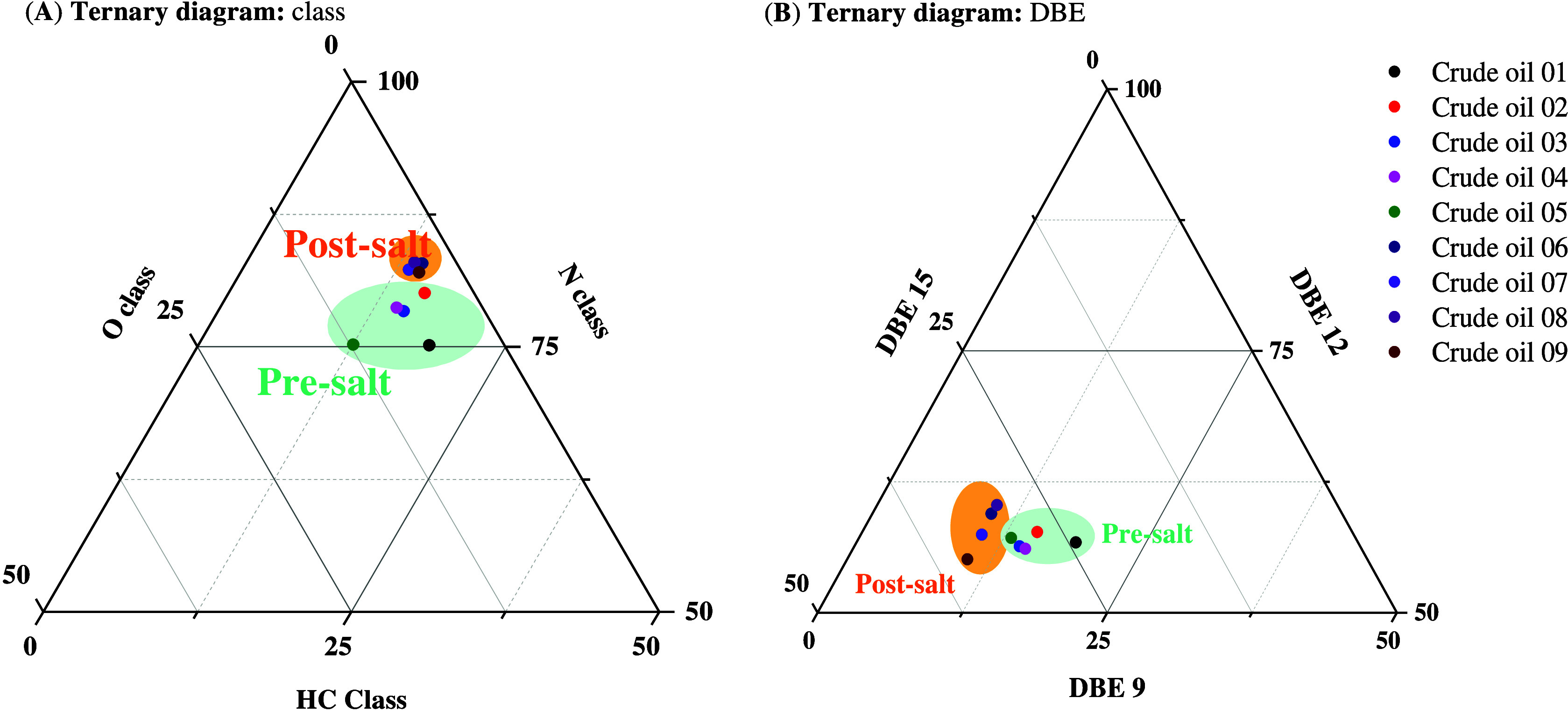
Ternary diagrams illustrating the molecular differentiation between presalt and postsalt crude oil samples based on (**A**) compound class distribution (HC, N, and O classes) and (**B**) relative abundances of hydrocarbon species with DBE 9, 12, and 15. Data were obtained by ESI (−) Orbitrap MS using 3% TMAH as dopant. Pre- and postsalt oils correspond to crude oils **01**–**05** and crude oils **06**–**09**, respectively.

A more distinctive geochemical signal emerged when the analysis was directed toward the HC class and its DBE distribution. In particular, the relative abundances of species with DBE of 9, 12, and 15, attributed to fluorene, benzofluorene, and dibenzofluorene, respectively, enabled further stratification of the samples as illustrated in Figure 4 (**B**). Presalt oils exhibited higher proportions of DBE 12 and 15 species, whereas postsalt oils were enriched in DBE 9 compounds. This distribution is consistent with known geochemical trends, where higher DBE fluorenes are associated with terrestrial organic matter, and DBE 9 species are linked to marine inputs.

Fluorene and its benzo-derivatives are well-established molecular markers in oil geochemistry due to their formation via diagenetic transformation of biphenyl structures within source rocks. Their abundance and alkylation patterns are sensitive indicators of the depositional environment and kerogen type. Terrestrial-derived inputs tend to yield more extensively alkylated (higher DBE) fluorenes, while marine-dominated systems produce lower-alkylated homologues.^56^ Consequently, the DBE profile within the HC class not only reflects the molecular diversity accessible through TMAH-assisted ESI but also provides a robust framework for oil-to-oil and oil-to-source correlation.

In summary, TMAH-assisted ESI (−) proved to be an effective and accessible strategy for enhancing the ionization of sp^3^-rich PAHs, enabling the detection of low-polarity and weakly acidic compounds often overlooked by conventional methods. Applied here in a petroleomic context, this approach provided deeper molecular insight into crude oil composition and source-related differences, demonstrating its broader applicability to complex sample analysis across environmental and geochemical fields.

## Conclusions

This study demonstrates the analytical advantages of employing TMAH as a dopant in ESI for the high-resolution mass spectrometric analysis of complex organic mixtures. By enhancing the ionization efficiency of weakly acidic and nonpolar compounds, particularly PAHs, TMAH effectively overcomes intrinsic limitations of conventional ESI. This enabled a substantial expansion of detectable chemical space, granting access to molecular classes that are typically underrepresented in direct ESI analyses.

In the context of petroleomics, TMAH-assisted ESI provided a robust platform for the detailed molecular characterization of crude oil samples. Beyond improving overall compositional coverage, the method allowed for refined structural interpretation of key hydrocarbon species, such as fluorene derivatives, without requiring derivatization or alternative ionization techniques. This enhanced sensitivity and selectivity translated into practical applications for differentiating petroleum reservoirs, showcasing the potential of TMAH-modified ionization strategies for source correlation and reservoir characterization.

Importantly, the benefits of TMAH-assisted ESI are not restricted to petroleum research. The approach offers a straightforward and broadly applicable strategy for improving the ionization of challenging analytes in various complex matrices. By expanding the analytical capabilities of ESI-MS, this methodology opens new possibilities for molecular-level investigations across multiple research fields, from geochemistry to energy, materials science, and beyond.

## Supplementary Material



## Notes

Additional experimental details, including physical-chemical characterization of crude oil samples; negative-ion mode ESI Orbitrap mass spectra using different dopants; and DBE and carbon number distributions for key heteroatomic classes and PAHs in pre- and postsalt crude oil samples (PDF)

## Abbreviations

APCI: Atmospheric Pressure Chemical Ionization
APPI: Atmospheric Pressure Photoionization
BA: Benzoic Acid
BC: Benzo­[*a*]­carbazole
CyPh: 4H-Cyclopenta­[*d*, *e*, *f*]­phenanthrene
DBE: Double Bond Equivalent
DBT: Dibenzothiophene
ESI: Electrospray Ionization
FT-ICR MS: Fourier Transform Ion Cyclotron Resonance Mass Spectrometry
fwhm: Full Width At Half Maximum
HESI: Heated Electrospray Ionization
HRMS: High-Resolution Mass Spectrometry
PAHs: Polycyclic Aromatic Hydrocarbons
SA: Stearic Acid
TMAH: Tetramethylammonium Hydroxide
