# Supplementary material for: Unlocking PAH Ionization in Negative-Mode ESI Orbitrap MS Using Tetramethylammonium Hydroxide: A Petroleomic Strategy
Source: J Am Soc Mass Spectrom. 2025 Sep 17;36(10):2142–50. doi: 10.1021/jasms.5c00170 (PMC12492404; doi:10.1021/jasms.5c00170)
Supplement: Supplementary file 1 [file js5c00170_si_001.pdf]

Supporting Information

Unlocking PAH Ionization in Negative-Mode ESI Orbitrap MS Using Tetramethylammonium Hydroxide: A Petroleomic Strategy

Deborah V. A. de Aguiar [a], Lidya C. da Silva[a]*, Iris Medeiros Júnior [b], Alexandre de O. Gomes[b], Boniek Gontijo [a,*]

[a] Laboratory of Chromatography and Mass Spectrometry, Institute of Chemistry, Federal University of Goiás, Goiânia, 59078-970, GO, Brazil.

[b] CENPES, PETROBRAS, Rio de Janeiro, RJ, 21941-915, Brazil.

*Corresponding author.

E-mail address: boniek@ufg.br (B. Gontijo)

Table of contents:

Table S1. Physical-chemical properties of the crude oil samples analyzed by negative-ion mode ESI Orbitrap MS 2

Figure S1. Negative-ion mode ESI Orbitrap mass spectra of the crude oil (**A**) **01** (**B**) **03**, and (**C**) **09** obtained with an ammonium hydroxide-modified electrospray dopant (top) and a different concentration of TMAH modified electrospray dopant. 2

Figure S2. Color-mapped plots of DBE versus carbon number for N, N_2, NO, and O classes for crude oil (**A**) **01** (**B**) **03**, and (**C**) **09** analyzed by negative-ion mode ESI Orbitrap MS with ammonium hydroxide and TMAH at 3%. 3

Figure S3. Relative abundance-based class distributions for the (**A**) pre- and (**B**) post-salt crude oils samples analyzed by negative-ion mode ESI Orbitrap MS...................................4

Table S1. Physical-chemical properties of the crude oil samples analyzed by negative-ion mode ESI Orbitrap MS

**Sample**	**Source**	**API gravity**	**Non-basic nitrogen content (%, *m/m*)**	**Sulphur content (%, *m/m*)**	**TAN (mg KOH.g^{-1})**
**01**	Pre-salt	35.90	0.09	0.08	0.11
**02**	Pre-salt	28.60	0.20	0.32	0.14
**03**	Pre-salt	29.90	0.14	0.36	0.26
**04**	Pre-salt	29.90	0.15	0.35	0.22
**05**	Pre-salt	17.50	0.38	0.74	0.79
**06**	Post-salt	27.50	0.22	0.76	0.13
**07**	Post-salt	27.20	0.25	0.54	0.43
**08**	Post-salt	23.80	0.20	0.52	0.66
**09**	Post-salt	18.80	0.32	0.73	2.32

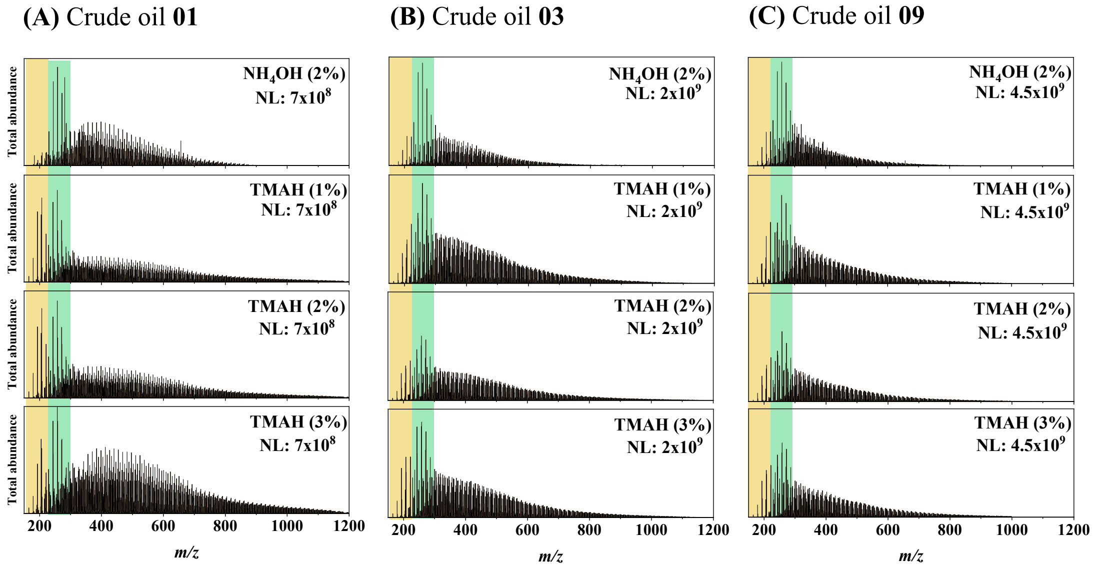

Figure S1. Negative-ion mode ESI Orbitrap mass spectra of the crude oil (**A**) **01** (**B**) **03**, and (**C**) **09** obtained with an ammonium hydroxide-modified electrospray dopant (top) and a different concentration of TMAH modified electrospray dopant.

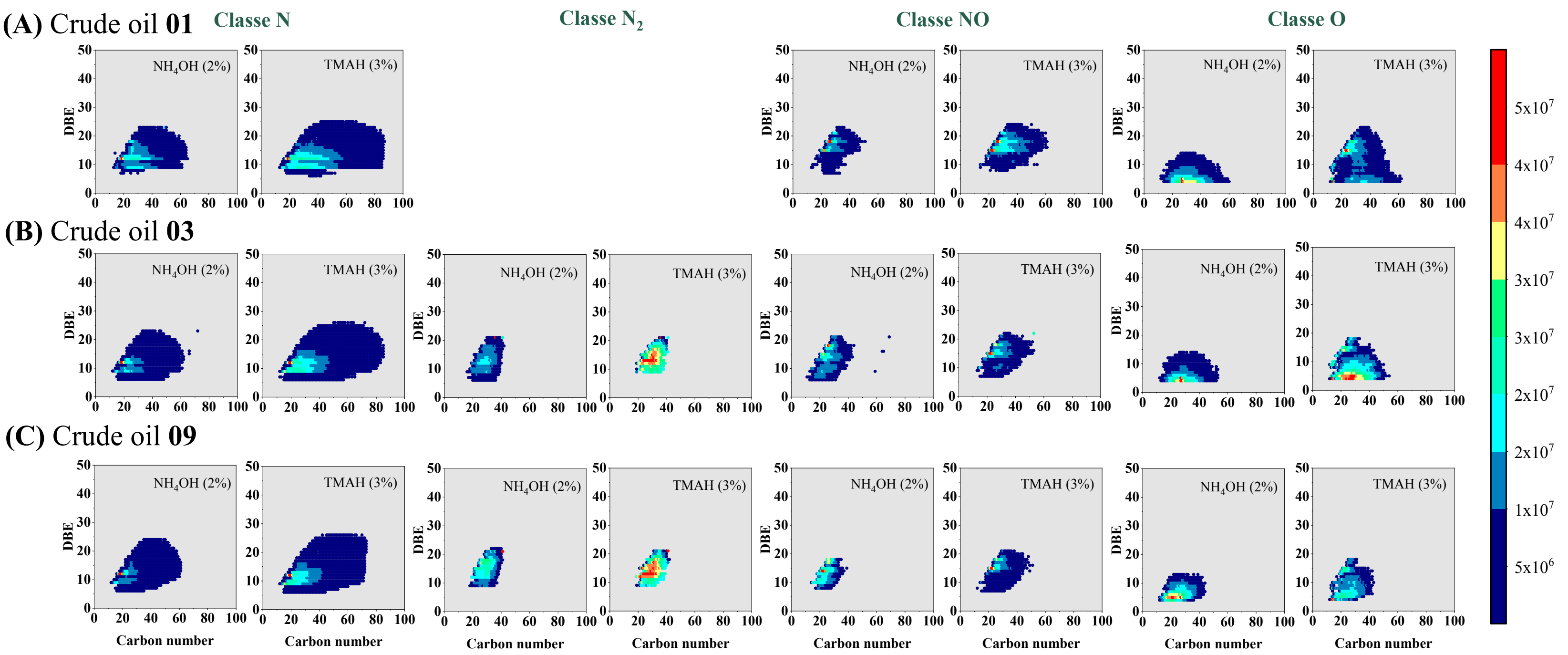

Figure S2. Color-mapped plots of DBE versus carbon number for N, N_2, NO, and O classes for crude oil (**A**) **01** (**B**) **03**, and (**C**) **09** analyzed by negative-ion mode ESI Orbitrap MS with ammonium hydroxide and TMAH at 3%.

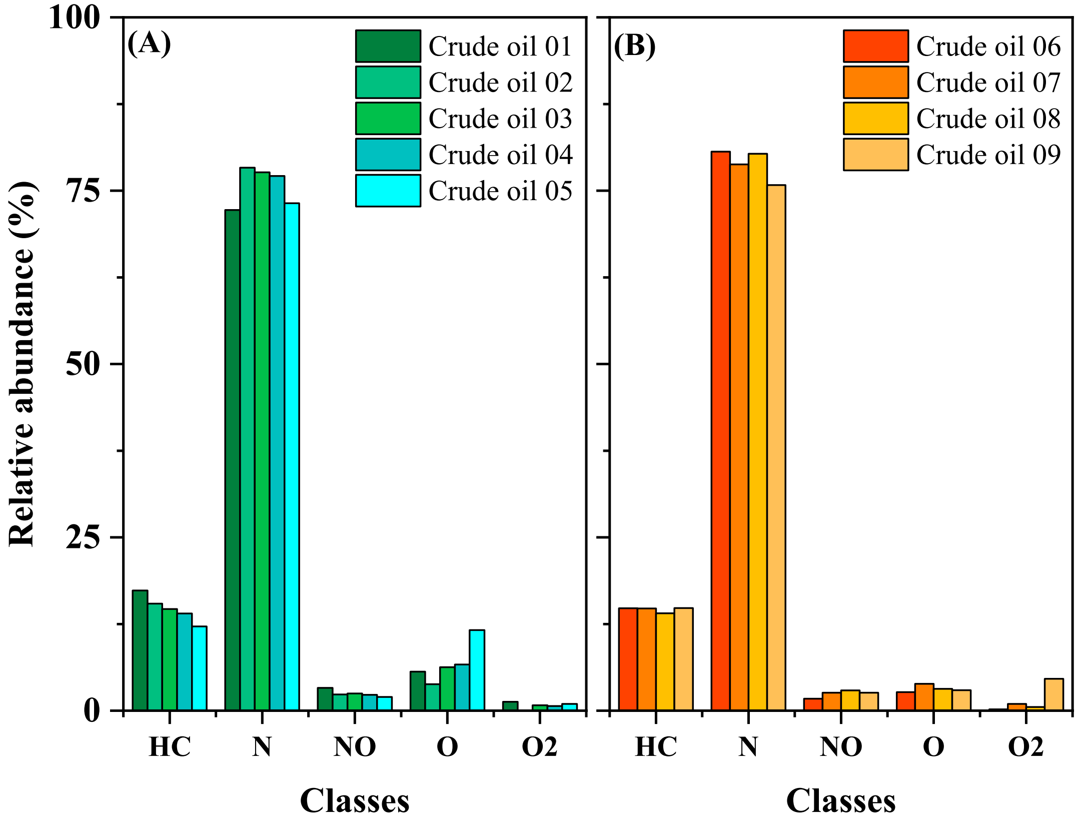

Figure S3. Relative abundance-based class distributions for the (**A**) pre- and (**B**) post-salt crude oils samples analyzed by negative-ion mode ESI Orbitrap MS.
